# Supplementary material for: Strategy and Performance Evaluation of Low-Frequency Variant Calling for SARS-CoV-2 Using Targeted Deep Illumina Sequencing
Source: Front Microbiol. 2021 Oct 13;12:747458. doi: 10.3389/fmicb.2021.747458 (PMC8548777; doi:10.3389/fmicb.2021.747458)
Supplement: Supplementary file 1 [file Data_Sheet_1.pdf]

Supplementary files

Supplementary File S1: List of initial 316 samples for which a consensus sequence was generated

Supplementary File S2: Scripts for LFV workflow

Lofreq_Workflow.smk

```
from pathlib import Path
import os
root = Path('Path/To/Samples')

all_samples = [fq.name.split('_')[0] for fq in root.iterdir() if '_R1_001.fastq.gz'
in fq.name]

print(all_samples)

snake_dir = workflow.basedir

rule all:
    input:
        CSV = expand(str(root / '{sample}' / 'lofreq' /  '{sample}_nofilter.csv'),
sample=all_samples),

rule trim_reads:
    """
    Trims paired end reads using trimmomatic.
    """
    input:
        FQ_fwd = lambda wildcards: str(root /
f'{wildcards.sample}_R1_001.fastq.gz'),
        FQ_rev = lambda wildcards: str(root /
f'{wildcards.sample}_R2_001.fastq.gz'),
    output:
        FQ_1P = temporary(root / '{sample}' / 'trimming' /
'trimmed_reads_1P.fastq'),
        FQ_2P = temporary(root / '{sample}' / 'trimming' /
'trimmed_reads_2P.fastq'),
        FQ_1U = temporary(root / '{sample}' / 'trimming' /
'trimmed_reads_1U.fastq'),
        FQ_2U = temporary(root / '{sample}' / 'trimming' /
'trimmed_reads_2U.fastq'),
    params:
        basename_output = lambda wildcards: root / wildcards.sample / 'trimming' /
'trimmed_reads.fastq',
        min_len = 40
    threads: 4
    shell:
        """
        module load trimmomatic/0.38;
        cd {params.basename_output.parent}; # Directory is changed because multiple
trimmomatic runs in the same directory interfere with each other
        trimmomatic.sh PE -baseout {params.basename_output} -threads {threads}
{input.FQ_fwd} {input.FQ_rev} \
        ILLUMINACLIP:$TRIMMOMATIC_ADAPTER_DIR/NexteraPE-PE.fa:2:30:10 LEADING:10
TRAILING:10 SLIDINGWINDOW:4:20 \
        MINLEN:{params.min_len}
        """
```

```
rule consensus_fasta_index:
    input:
        FASTA = lambda wildcards: str(root / f"{wildcards.sample.split('-
')[0]}.fasta")
    output:
        INDEX = root / '{sample}' / 'lofreq' / 'reference' / '{sample}.fasta'
    shell:
        """
        module load samtools/1.9;
        module load bowtie2/2.3.4.3;
        cp {input.FASTA} {output.INDEX};
        samtools faidx {output.INDEX};
        bowtie2-build {output.INDEX} {output.INDEX};
        """

rule bt2_map_reads:
    input:
        FASTA = rules.consensus_fasta_index.output.INDEX,
        FQ_1P = rules.trim_reads.output.FQ_1P,
        FQ_2P = rules.trim_reads.output.FQ_2P,
        FQ_1U = rules.trim_reads.output.FQ_1U,
        FQ_2U = rules.trim_reads.output.FQ_2U
    output:
        SAM = temporary(root / '{sample}' / 'lofreq' / '{sample}.sam')
    threads: 16
    shell:
        """
        module load bowtie2/2.3.4.3;
        bowtie2 -x {input.FASTA} -1 {input.FQ_1P} -2 {input.FQ_2P} -S {output.SAM}
-p {threads};
        """

rule sam_to_bam:
    input:
        SAM = rules.bt2_map_reads.output.SAM
    output:
        BAM = temporary(root / '{sample}' / 'lofreq' /  '{sample}.bam')
    shell:
         """
         module load samtools/1.9;
         samtools view -S -b {input.SAM} > {output.BAM};
                  """

rule sort_bam:
    input:
        BAM = rules.sam_to_bam.output.BAM
    output:
        SORT_BAM = temporary(root / '{sample}' / 'lofreq' / '{sample}_sort.bam'),
        SORT_BAM_index = root / '{sample}' / 'lofreq' /  '{sample}_sort.bam.bai'
    shell:
         """
         module load samtools/1.9;
         samtools sort -o {output.SORT_BAM} --output-fmt bam --threads 8
{input.BAM}
         samtools index {output.SORT_BAM};
         """

rule samtools_depth:
    input:
        SORTBAM = rules.sort_bam.output.SORT_BAM
```

```
    output:
        DEPTH = temporary(root / '{sample}' / 'lofreq' / '{sample}_Depth.txt')
    shell:
         """
         module load samtools/1.9;
         samtools depth -d 1000000 {input.SORTBAM} -aa | awk '{{print $1, $3}}' >
{output.DEPTH}
         """

rule median_average_depth:
    input:
        DEPTH = {rules.samtools_depth.output.DEPTH}
    output:
        AV_DEPTH = root / '{sample}' / 'lofreq' /
'{sample}_Depth_AverageCoverage.txt',
        MED_DEPTH = root / '{sample}' / 'lofreq' /
'{sample}_Depth_MedianCoverage.txt'
    params:
        base = lambda wildcards: wildcards.sample
    shell:
         """
         sort -V {input.DEPTH} | awk '{{a[i++]=$2; }} END {{print "{params.base}"
";" a[int(i/2)]; }}' > {output.MED_DEPTH}
         sort -V {input.DEPTH} | awk '{{total += $2 }} END {{print "{params.base}"
";" total/NR; }}' > {output.AV_DEPTH}
         """
rule bam_read_count:
    input:
        MED_DEPTH = rules.median_average_depth.output.MED_DEPTH,
        SORTBAM = rules.sort_bam.output.SORT_BAM,
        FASTA = rules.consensus_fasta_index.output.INDEX,
    output:
        TXT = root / '{sample}' / 'lofreq' / '{sample}_bamreadcount.txt'
    params:
        script = 'bam-readcount/bin/bam-readcount',
    resources:
        mem_mb=80000
    shell:
         """
         var=$(grep -e "^>" {input.FASTA} | awk 'sub(/^>/, "")')
         {params.script} -f {input.FASTA} {input.SORTBAM} -w 1 -d 10000000 $var:1-
29861 > {output.TXT}
         """

rule create_seq_dict:
    input:
        TXT = rules.bam_read_count.output.TXT,
        FASTA = rules.consensus_fasta_index.output.INDEX,
    output:
        DICT = root / '{sample}' / 'lofreq' / 'reference' / '{sample}.dict'
    shell:
         """
         module load picard/2.18.14;
         run_picard.sh CreateSequenceDictionary REFERENCE={input.FASTA}
OUTPUT={output.DICT} ;
         """

rule sort_sam_picard:
    input:
        BAMREADCOUNT = {rules.bam_read_count.output.TXT},
        BAM = rules.sam_to_bam.output.BAM,
```

```
        DICT = rules.create_seq_dict.output.DICT
    output:
        SortBAM = temporary(root / '{sample}' / 'lofreq' /
'{sample}_picardSorted.bam')
    shell:
         """
         module load picard/2.18.14;
         run_picard.sh SortSam I={input.BAM} O={output.SortBAM}
SORT_ORDER=coordinate
         """


rule index_picardSorted_bam:
    input:
        Dedup_BAM = rules.sort_sam_picard.output.SortBAM
    output:
        index_Dedup_BAM = root / '{sample}' / 'lofreq' /
'{sample}_picardSorted.bam.bai',
    shell:
         """
         module load samtools/1.9;
         samtools index {input.Dedup_BAM}
         """

rule read_groups:
    input:
        Dedup_BAM = rules.sort_sam_picard.output.SortBAM,
        index_Dedup_BAM = rules.index_picardSorted_bam.output.index_Dedup_BAM
    output:
        gp_BAM = temporary(root / '{sample}' / 'lofreq' /  '{sample}_gp.bam'),
    shell:
         """
         module load picard/2.18.14;
         run_picard.sh AddOrReplaceReadGroups I={input.Dedup_BAM} O={output.gp_BAM}
LB=test PL=test PU=test SM=test
         """

rule index_gp_bam:
    input:
        GP_BAM = rules.read_groups.output.gp_BAM
    output:
        index_gp_BAM = root / '{sample}' / 'lofreq' /  '{sample}_gp.bam.bai',
    shell:
         """
         module load samtools/1.9;
         samtools index {input.GP_BAM}
         """

rule realigner_targetcreator:
    input:
        FASTA = rules.consensus_fasta_index.output.INDEX,
        GP_BAM = rules.read_groups.output.gp_BAM,
        index_Dedup_BAM = rules.index_gp_bam.output.index_gp_BAM,
        DICT = rules.create_seq_dict.output.DICT
    output:
        INTERVAL = root / '{sample}' / 'lofreq' /  '{sample}.intervals',
    shell:
         """
         module load gatk/3.7;
         run_gatk.sh -T RealignerTargetCreator -R {input.FASTA} -I {input.GP_BAM} -
o {output.INTERVAL};
```

```
         """

rule indel_realigner:
    input:
        FASTA = rules.consensus_fasta_index.output.INDEX,
        GP_BAM = rules.read_groups.output.gp_BAM,
        INTERVAL = rules.realigner_targetcreator.output.INTERVAL
    output:
        REAL_BAM = temporary(root / '{sample}' / 'lofreq' /  '{sample}_real.bam'),
    shell:
         """
         module load gatk/3.7;
         run_gatk.sh  -T IndelRealigner -maxReads 1000000 -R {input.FASTA} -I
{input.GP_BAM} -targetIntervals {input.INTERVAL} -o {output.REAL_BAM};
         """

rule index_real_bam:
    input:
        REAL_BAM = rules.indel_realigner.output.REAL_BAM
    output:
        index_real_BAM = root / '{sample}' / 'lofreq' /  '{sample}_real.bam.bai',
    shell:
         """
         module load samtools/1.9;
         samtools index {input.REAL_BAM}
         """

rule lofreq_indelqual:
    input:
        FASTA = rules.consensus_fasta_index.output.INDEX,
        REAL_BAM = rules.indel_realigner.output.REAL_BAM,
        INTERVAL = rules.realigner_targetcreator.output.INTERVAL,
        INDEX = rules.index_real_bam.output.index_real_BAM
    output:
        INDELQUAL_BAM = temporary(root / '{sample}' / 'lofreq' /
'{sample}_indelqual.bam'),
    shell:
         """
         module load lofreq_star/2.1.3.1;
         lofreq indelqual --dindel -f {input.FASTA} -o {output.INDELQUAL_BAM}
{input.REAL_BAM}
         """

rule lofreq_indelqual_index:
    input:
        indelqual_BAM = rules.lofreq_indelqual.output.INDELQUAL_BAM
    output:
        index_indelqual_BAM = root / '{sample}' / 'lofreq' /
'{sample}_indelqual.bam.bai',
    shell:
         """
         module load samtools/1.9;
         samtools index {input.indelqual_BAM}
         """

rule lofreq_call:
    input:
        FASTA = rules.consensus_fasta_index.output.INDEX,
        INDELQUAL_BAM = rules.lofreq_indelqual.output.INDELQUAL_BAM,
        index_INDELQUAL_BAM =
rules.lofreq_indelqual_index.output.index_indelqual_BAM
```

```
    output:
        LOFREQ_VCF = root / '{sample}' / 'lofreq' /  '{sample}_unfiltered.vcf',
    shell:
         """
         module load lofreq_star/2.1.3.1;
         lofreq call --call-indels --no-default-filter -f {input.FASTA} -o
{output.LOFREQ_VCF} {input.INDELQUAL_BAM}
         """

rule lofreq_filter:
    input:
        LOFREQ_VCF = rules.lofreq_call.output.LOFREQ_VCF
    output:
        LOFREQ_VCF_2 = root / '{sample}' / 'lofreq' /  '{sample}_filtered.vcf',
    shell:
         """
         module load lofreq_star/2.1.3.1;
         lofreq filter -i {input.LOFREQ_VCF} -o {output.LOFREQ_VCF_2} --sb-thresh
2147483647
         """

rule add_info_to_vcf:
    input:
        REAL_BAM = rules.indel_realigner.output.REAL_BAM,
        LOFREQ_VCF_2 = rules.lofreq_filter.output.LOFREQ_VCF_2
    output:
        POS_Q_VCF_2 = root / '{sample}' / 'lofreq' /  '{sample}_pos_q.vcf'
    params:
        script = os.path.join(snake_dir, "../python/scripts/add_info_to_vcf.py")
    shell:
         """
         python {params.script} {input.REAL_BAM} {input.LOFREQ_VCF_2}
{output.POS_Q_VCF_2}
         """

rule vcf_to_csv:
    input:
        POS_Q_VCF_2 = rules.add_info_to_vcf.output.POS_Q_VCF_2
    output:
        CSV_nF_2 = root / '{sample}' / 'lofreq' /  '{sample}_nofilter.csv'
    params:
        script = os.path.join(snake_dir, "../python/scripts/vcf_to_csv_lofreq.py"),
        base = lambda wildcards: wildcards.sample
    shell:
         """
         python {params.script} -I {input.POS_Q_VCF_2} -O {output.CSV_nF_2} -S
{params.base} -F none
         """
```

1 add_info_to_vcf.py

```
# modified from lauringlab (cf
https://github.com/lauringlab/Benchmarking_paper/blob/master/scripts/mapq_vcf.py)
#!/usr/bin/python

import numpy as np
import pysam
import os
import vcf
from vcf.parser import _Info as VcfInfo
import sys


def filter(bam_file=None, in_vcf_file=None, out_vcf_file=None):
    """
    Doesn't filter, per say.
    Adds average phred scores, read positions and MapQ.
    :param bam_file:
    :param in_vcf_file:
    :param out_vcf_file:
    :return:
    """

    # Not useful, it seems.
    # input = in_vcf_file
    # if "/" in input:
    #     input = input.split("/")[-1]
    #
    # input=input.split(".")[0]

    in_var = vcf.Reader(open(in_vcf_file, 'r'))
    ## update infos ##
    in_var.infos['MapQ']=VcfInfo(id='MapQ',num=1,type='Float',desc="The average
MapQ of the reads containing the called variant", source=None, version=None)
    in_var.infos['Read_pos']=VcfInfo(id='Read_pos',num=1,type='Float',desc="The
average read cycle that called the given variant", source=None, version=None)
    in_var.infos['Phred']=VcfInfo(id='Phred',num=1,type='Float',desc="The average
Phred score of the called variant", source=None, version=None)

    variants=list(in_var)
    if len(variants) != 0:
        with pysam.AlignmentFile(bam_file, "rb") as bamfile:

            for record in variants:
                # TD for debugging
                print(record)
                print(record.POS)
                if record.POS == "1026":
                    pass
                mapq=[]  # This will hold a list of the mapping qualities that map
to the variant
                phred=[]  # This will hold a list of the phred that map to the
variant
                Read_pos = []  # This will hold a list of position relative to the
read
                chr=record.CHROM
                pos=int(record.POS)
                py_pos=pos-1
```

```
                var=record.ALT[0]
                # record.ID=input
                #stepper="nofilter", min_base_quality=0,  doesn't change anything
on 1 dataset tested
                for pileupcolumn in
bamfile.pileup(chr,py_pos,py_pos+1,truncate=True,stepper="all", max_depth=1E6):
                    if pileupcolumn.pos==py_pos:
                        for pileupread in pileupcolumn.pileups:
                            if not pileupread.is_del and not pileupread.is_refskip:

called_base=pileupread.alignment.query_sequence[pileupread.query_position]

called_phred=pileupread.alignment.query_qualities[pileupread.query_position]
                                if called_phred>0 and called_base==var: # change
this if you change the phred cut off in deepSNV

mapq.append(pileupread.alignment.mapping_quality)
                                    phred.append(called_phred)
                                    Read_pos.append(pileupread.query_position)
                mean_map=np.mean(mapq)
                mean_phred=np.mean(phred)
                mean_Read_pos=np.mean(Read_pos)

                if mean_map==[]:
                    print( "OOPS didn't find the variant looks like you didn't fix
the bug")
                    sys.exit(1)

                record.add_info('MapQ',mean_map)
                record.add_info('Read_pos',mean_Read_pos)
                record.add_info('Phred',mean_phred)

        print ("done updating")
        iter(variants)
        vcf_writer = vcf.Writer(open(out_vcf_file, 'w'), in_var)
        for record in variants:
            vcf_writer.write_record(record)
        vcf_writer.close()

    else:
        print("No var provided")

def main():
    args = sys.argv[:]
    if len(args) != 4:
        sys.exit("Not all required arguments were present. Exiting.")

    bam_file = args[1]
    in_vcf_file = args[2]
    out_vcf_file = args[3]

    if not os.path.isfile(out_vcf_file):
        if not os.path.isdir(os.path.split(out_vcf_file)[0]):
            os.mkdir(os.path.split(out_vcf_file)[0])
        # print(sample)
        # print(in_vcf_file)
        filter(bam_file, in_vcf_file, out_vcf_file)

if __name__ == main():
    main()
```

2 vcf_to_csv_lofreq.py

```
import csv
import os
import vcf
import sys
import traceback
import argparse


def parse_cla():

    ap = argparse.ArgumentParser()
    ap.add_argument('-O', '--output_csv_path', dest='out_csv_file',
metavar='out_csv_file', required=True, help="Absolute path to output csv file.")
    ap.add_argument('-I', '--in_vcf_path', dest='in_vcf_path',
metavar='in_vcf_path', required=True, help="Absolute path to input vcf file")
    ap.add_argument('-S', '--sample_name', dest='sample_name',
metavar='sample_name', required=True, help="Sample name to be added to each variant
line. Default sampleX", default="sampleX")
    ap.add_argument('-F', '--filter', dest='filter', metavar="filter",
choices=["none", "position", "AF"], help="Filtering to perform; either none (all
variants added to csv), position (only variants within specified average reads
positions are kept), or AF (NOT IMPLEMENTED - filters out based on AF of variant)",
required = True)
    ap.add_argument('-mirp', '--min_read_pos', dest='min_read_pos',
metavar='min_read_pos', required=False, help="Minimal average read position for
filtering on read positions. Required if using --filter position . Default 62.",
default=62)
    ap.add_argument('-marp', '--max_read_pos', dest='max_read_pos',
metavar='max_read_pos', required=False,
                    help="Max average read position for filtering on read
positions. Required if using --filter position . Default 188.",
                    default=188)
    return ap.parse_args()


def validate_args(args=None):
    """
    Check that all provided arguments are correct and coherent, expected files
exist, ....
    :param args:
    :return:
    """
    validated = True

    if args is None:
        print("No args.")
        validated = False
    else:
        if not os.path.isfile(args.in_vcf_path):
            print(f"No vcf file for {args.in_vcf_path}.")
            validated = False

        if args.filter == "AF":
            print(f"filter_high_fq not implemented yet")
            validated = False

    if not validated:
        print(f"Exiting.")
```

```
        sys.exit(1)


def print_pre_run_info(args=None):
    """
    Print warnings and info before running based on args.
    :return:
    """
    print(f"Running with min_read_pos={args.min_read_pos} and max_read_pos =
{args.max_read_pos}.")


def filter_vcf_file(min_read_pos=None, max_read_pos=None, in_vcf_file_path=None,
filter=None):
    """
    Filter the records in vcf file and output the kept records as a list.
    :return: list
    """
    results = []

    with open(in_vcf_file_path, 'r') as in_vcf_file_stream:
        vcf_reader = vcf.Reader(in_vcf_file_stream)
        for record in vcf_reader:
            output_record = [record.CHROM, record.POS, record.REF, record.ALT,
record.INFO["AF"], record.INFO["DP"], record.INFO["SB"]]
            add_record_to_output = False
            try:

                if filter == "none":
                    add_record_to_output = True

                # filter-out extreme average read positions
                elif filter == "position":
                    if min_read_pos < record.INFO['Read_pos'] < max_read_pos:
                        add_record_to_output = True

                # filter on AF values
                elif filter == "AF":
                    pass

                if add_record_to_output:
                    results.append(output_record)

            except:
                print("Error with the following variant:")
                print(output_record)
                print(traceback.format_exc())

    return results

def write_csv(output_csv_path=None, results=None, sample_name=None):
    """
    Write csv file with output records.
    :return:
    """

    out_header = ["Sample", "CHROM", "POS", "REF", "ALT", "VF", "DP", "SB"]

    with open(output_csv_path, "w") as out_csv_stream:
        csv_writer = csv.writer(out_csv_stream)
        csv_writer.writerow(out_header)
```

```
        for record in results:
            line = [sample_name]
            for elem in record:
                line.append(elem)
            csv_writer.writerow(line)

def main():

    args = parse_cla()

    validate_args(args=args)
    print_pre_run_info(args=args)

    in_vcf_file_path = args.in_vcf_path
    output_csv_path = args.out_csv_file
    sample_name = args.sample_name

    filter = args.filter
    min_read_pos = args.min_read_pos
    max_read_pos = args.max_read_pos

    results = filter_vcf_file(min_read_pos=min_read_pos, max_read_pos=max_read_pos,
in_vcf_file_path=in_vcf_file_path, filter=filter)

    write_csv(output_csv_path=output_csv_path, results=results,
sample_name=sample_name)


main()
```

Supplementary Table S1: Coverages of the positions characteristic to B.1.1.7 mutations per sample after normalization. The cells are coloured according to their group, either with a coverage of approximately 5000X (yellow) or approximately 10,000X (green)

	Mutant										Wild-type									
	ERR5059072	ERR5059238	ERR5059260	ERR5059092	ERR5059204	ERR5059123	ERR5059282	ERR5058968	ERR5059226	ERR5059033	ERR5059114	ERR5059253	ERR5059286	ERR5059283	ERR5039162	ERR5059083	ERR5059133	ERR5059257	ERR5059154	ERR5040499
**C913T**	4917	4881	4845	4780	4856	4837	4813	4921	4850	4745	4835	4862	4839	4864	4929	4926	4910	4828	4880	4884
**C3267T**	4849	4779	4839	4814	4853	4919	4864	4883	4816	4870	4886	4891	4786	4909	4781	4896	4859	4863	4839	4793
**C5388A**	4790	4809	4768	4767	4886	4869	4862	4803	4767	4727	4867	4784	4798	4902	4821	4862	4861	4716	4811	3580
**C5986T**	4783	4863	4878	4788	4921	4940	4883	4778	4872	4769	4841	4904	4885	4869	4907	4905	4908	4847	4912	4807
**T6954C**	4793	4868	4846	4746	4850	4800	4839	4823	4874	4724	4821	4925	4849	4828	4912	4856	4835	4844	4872	4563
**11288-11296 deletion**	5015	4980	4962	4904	5007	4972	5034	4879	4965	4944	5009	4936	4992	5001	5008	5040	4962	4988	4973	4858
**C14676T**	4813	4825	4764	4715	4834	4794	4734	4861	4828	4633	4780	4836	4821	4763	4804	4848	4870	4835	4828	4731
**C15279T**	4787	4770	4777	4722	4807	4831	4798	4815	4798	4722	4877	4789	4790	4765	4855	4800	4795	4797	4807	4796
**T16176C**	9712	9883	9900	9489	9734	9796	9783	9738	9871	9627	8998	9892	9855	9855	9695	9817	9722	9806	9898	9543
**21765-21770 deletion**	4846	4816	4894	4729	4835	4833	4795	4797	4845	4688	4882	4906	4803	4794	4799	4841	4813	4769	4882	4718
**21991-21993 deletion**	9718	9778	9759	9552	9728	9753	9766	9748	9703	9494	9823	9827	9774	9827	9796	9723	9773	9741	9850	9636
**A23063T**	4459	4912	5049	4862	4839	4852	4899	4886	4896	4571	4916	4915	4867	4928	4872	4378	4458	4923	4883	4759
**C23271A**	4730	4835	4713	4636	4735	4823	4859	4782	4772	4694	4786	4830	4811	4889	4776	4825	4840	4796	4755	4644
**C23604A**	4807	4869	4878	4766	4913	4824	4822	4826	4900	4710	4843	4803	4860	4842	4768	4868	4813	4796	4932	4761
**C23709T**	4882	4904	4907	4901	4952	4846	4853	4852	4929	4878	4868	4840	4885	4869	4799	4896	4846	4839	4961	4781
**T24506G**	4897	4900	4969	4925	4970	4882	4847	4847	4976	4932	4868	4827	4869	4807	4830	4895	4975	4949	4982	4814
**G24914C**	9428	9461	9370	9244	9519	9324	9458	9272	9403	9063	9525	9566	9460	9527	9536	9545	9387	9502	9459	9223
**G26801C**	4824	4829	4834	4732	4805	4837	4903	4854	4902	4669	4803	4818	4830	4870	4821	4873	4870	4860	4843	4770
**C27972T**	4900	4776	4851	4842	4833	4868	4780	4808	4877	4730	9325	9507	9097	9310	9304	9253	9292	9311	9505	7143
**G28048T**	4879	4765	4862	4750	4810	4888	4759	4817	4888	4679	4920	4969	4791	4882	4868	4888	4898	4876	4880	3787
**A28111G**	9626	9671	9729	9500	9655	9744	9677	9653	9720	9499	9719	9854	9661	9767	9687	9818	9852	9817	9705	8570
**G28280C**	11561	10638	10424	10081	10946	10522	10627	10569	10227	10228	10076	10285	10275	9736	10389	10492	10464	10299	10080	10033
**A28281T**	11563	10638	10424	10080	10946	10521	10626	10568	10225	10230	10077	10286	10276	9736	10389	10492	10464	10300	10079	10036
**T28282A**	11572	10638	10423	10080	10947	10520	10625	10568	10223	10233	10078	10289	10276	9736	10390	10493	10464	10300	10080	10037
**C28977T**	4864	4813	4845	4787	4925	4914	4833	4859	4835	4722	4824	4953	4853	4885	4782	4793	4922	4780	4897	4780

Supplementary Table S2: Number of mutations that were considered at which theoretical and median coverage. If N=7, it indicates that only the group of mutations with an initial coverage of approximately 10,000X was considered. If N=17, only the group of mutations with an initial coverage of approximately 5000X was considered. If N=24, it means that at that coverage all mutations could be considered.

Number of mutations	Theoretical coverage	Median coverage
N=7	10,000	9792
N=7	9000	8790
N=7	8000	7801
N=7	7000	6834
N=7	6000	5855
N=24	5000	4855
N=17	4500	4362
N=24	4000	3876
N=17	3500	3386
N=24	3000	2907
N=17	2500	2416
N=24	2000	1939.5
N=24	1500	1456
N=24	1000	971
N=17	750	730
N=24	500	483
N=17	250	239
N=7	200	201
N=17	100	98

Supplementary Figure S1: Qualitative evaluation of Dataset 1 using the number of false negatives divided by the number of observations per condition until a targeted mutant AF of 100%. Orange and red dots represent conditions with a FN proportion between 0 and 0.1, and between 0.1 and 1, respectively. The percentage of false negatives is coloured ranging from 0 (dark) to 1 (yellow) in intervals of 0.1 as extrapolated using a contour plot in the R package plotly [61] (actual FN proportions are presented in Supplementary Table S3). Note that a targeted AF of 0% corresponds to the wild-type and is therefore not presented. Both the x- and y-axis follow a logarithmic scale.

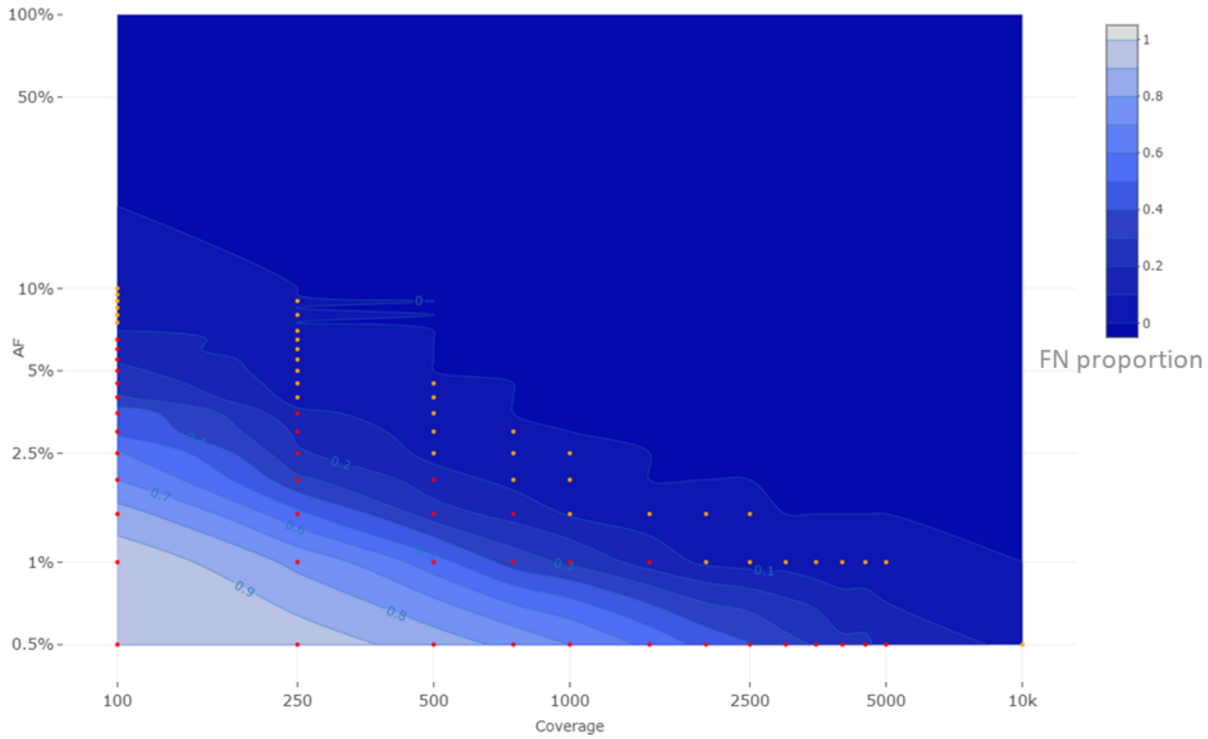

Supplementary Table S3: Qualitative evaluation of Dataset 1 using the number of false negatives divided by the number of observations per condition until a targeted mutant AF of 100%. The percentage of false negatives is coloured ranging from 0 (dark) to 1 (light) according to the gradient depicted in Supplementary Figure S1. Actual FN proportions are presented in Table 3. Note that a targeted AF of 0% corresponds to the wild-type and it is therefore not presented.

**Coverage →**<br>**AF ↓**	100	250	500	750	1000	1500	2000	2500	3000	3500	4000	4500	5000	10,000
100.0%	0%	0%	0%	0%	0%	0%	0%	0%	0%	0%	0%	0%	0%	0%
50.0%	0%	0%	0%	0%	0%	0%	0%	0%	0%	0%	0%	0%	0%	0%
40.0%	0%	0%	0%	0%	0%	0%	0%	0%	0%	0%	0%	0%	0%	0%
30.0%	0%	0%	0%	0%	0%	0%	0%	0%	0%	0%	0%	0%	0%	0%
20.0%	0%	0%	0%	0%	0%	0%	0%	0%	0%	0%	0%	0%	0%	0%
10.0%	5%	0%	0%	0%	0%	0%	0%	0%	0%	0%	0%	0%	0%	0%
9.5%	4%	0%	0%	0%	0%	0%	0%	0%	0%	0%	0%	0%	0%	0%
9.0%	7%	1%	0%	0%	0%	0%	0%	0%	0%	0%	0%	0%	0%	0%
8.5%	4%	0%	0%	0%	0%	0%	0%	0%	0%	0%	0%	0%	0%	0%
8.0%	9%	2%	0%	0%	0%	0%	0%	0%	0%	0%	0%	0%	0%	0%
7.5%	8%	0%	0%	0%	0%	0%	0%	0%	0%	0%	0%	0%	0%	0%
7.0%	10%	1%	0%	0%	0%	0%	0%	0%	0%	0%	0%	0%	0%	0%
6.5%	15%	2%	0%	0%	0%	0%	0%	0%	0%	0%	0%	0%	0%	0%
6.0%	15%	1%	0%	0%	0%	0%	0%	0%	0%	0%	0%	0%	0%	0%
5.5%	19%	3%	0%	0%	0%	0%	0%	0%	0%	0%	0%	0%	0%	0%
5.0%	22%	3%	0%	0%	0%	0%	0%	0%	0%	0%	0%	0%	0%	0%
4.5%	26%	4%	2%	0%	0%	0%	0%	0%	0%	0%	0%	0%	0%	0%
4.0%	31%	6%	1%	0%	0%	0%	0%	0%	0%	0%	0%	0%	0%	0%
3.5%	45%	12%	4%	0%	0%	0%	0%	0%	0%	0%	0%	0%	0%	0%
3.0%	47%	18%	4%	1%	0%	0%	0%	0%	0%	0%	0%	0%	0%	0%
2.5%	62%	21%	7%	2%	2%	0%	0%	0%	0%	0%	0%	0%	0%	0%
2.0%	70%	32%	14%	7%	3%	0%	0%	0%	0%	0%	0%	0%	0%	0%
1.5%	84%	52%	24%	16%	9%	5%	1%	2%	0%	0%	0%	0%	0%	0%
1.0%	96%	77%	54%	35%	28%	15%	8%	6%	6%	3%	2%	2%	2%	0%
0.5%	98%	95%	85%	77%	70%	57%	46%	41%	33%	29%	22%	22%	16%	7%
0.0%	0%	0%	0%	0%	0%	0%	0%	0%	0%	0%	0%	0%	0%	0%

Supplementary Figure S2: Qualitative evaluation of Dataset 2 using the number of false negatives divided by the number of observations per condition until a targeted mutant AF of 100%. Orange and red dots represent conditions with a FN proportion between 0 and 0.1, and between 0.1 and 1, respectively. The percentage of false negatives is coloured ranging from 0 (dark) to 1 (light) in intervals of 0.1 as extrapolated using a contour plot in the R package plotly [61] (actual FN proportions are presented in Supplementary Table S4). Note that a targeted AF of 0% represents to the wild-type and is therefore not presented. Both the x- and y-axis follow a logarithmic scale.

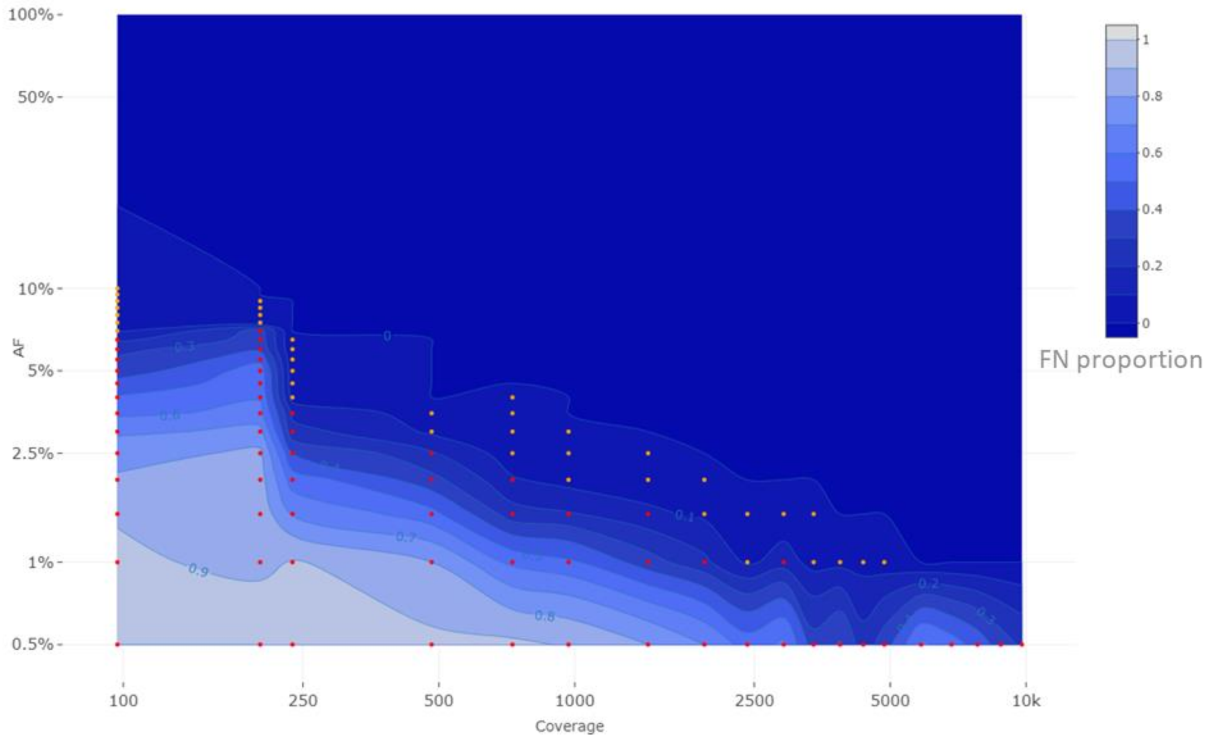

Supplementary Table S4: Qualitative evaluation of Dataset 2 using the number of false negatives divided by the number of observations per condition until a targeted mutant AF of 100%. The percentage of false negatives is coloured ranging from 0 (dark) to 1 (light) according to the gradient depicted in Supplementary Figure S2. Actual FN proportions are presented in Table 3. Note that a targeted AF of 0% corresponds to the wild-type and it is therefore not presented.

Coverage → AF ↓	97	201	237	482	728	969	1454	1937	2413	2904	3383	3872	4358	4851	5855	6834	7801	8790	9792
100.0%	0%	0%	0%	0%	0%	0%	0%	0%	0%	0%	0%	0%	0%	0%	0%	0%	0%	0%	0%
50.0%	0%	0%	0%	0%	0%	0%	0%	0%	0%	0%	0%	0%	0%	0%	0%	0%	0%	0%	0%
40.0%	0%	0%	0%	0%	0%	0%	0%	0%	0%	0%	0%	0%	0%	0%	0%	0%	0%	0%	0%
30.0%	0%	0%	0%	0%	0%	0%	0%	0%	0%	0%	0%	0%	0%	0%	0%	0%	0%	0%	0%
20.0%	0%	0%	0%	0%	0%	0%	0%	0%	0%	0%	0%	0%	0%	0%	0%	0%	0%	0%	0%
10.0%	2%	0%	0%	0%	0%	0%	0%	0%	0%	0%	0%	0%	0%	0%	0%	0%	0%	0%	0%
9.5%	3%	0%	0%	0%	0%	0%	0%	0%	0%	0%	0%	0%	0%	0%	0%	0%	0%	0%	0%
9.0%	5%	1%	0%	0%	0%	0%	0%	0%	0%	0%	0%	0%	0%	0%	0%	0%	0%	0%	0%
8.5%	6%	1%	0%	0%	0%	0%	0%	0%	0%	0%	0%	0%	0%	0%	0%	0%	0%	0%	0%
8.0%	8%	1%	0%	0%	0%	0%	0%	0%	0%	0%	0%	0%	0%	0%	0%	0%	0%	0%	0%
7.5%	8%	1%	0%	0%	0%	0%	0%	0%	0%	0%	0%	0%	0%	0%	0%	0%	0%	0%	0%
7.0%	9%	34%	0%	0%	0%	0%	0%	0%	0%	0%	0%	0%	0%	0%	0%	0%	0%	0%	0%
6.5%	18%	35%	1%	0%	0%	0%	0%	0%	0%	0%	0%	0%	0%	0%	0%	0%	0%	0%	0%
6.0%	28%	38%	1%	0%	0%	0%	0%	0%	0%	0%	0%	0%	0%	0%	0%	0%	0%	0%	0%
5.5%	31%	47%	3%	0%	0%	0%	0%	0%	0%	0%	0%	0%	0%	0%	0%	0%	0%	0%	0%
5.0%	35%	56%	3%	0%	0%	0%	0%	0%	0%	0%	0%	0%	0%	0%	0%	0%	0%	0%	0%
4.5%	43%	57%	4%	0%	0%	0%	0%	0%	0%	0%	0%	0%	0%	0%	0%	0%	0%	0%	0%
4.0%	51%	59%	6%	0%	1%	0%	0%	0%	0%	0%	0%	0%	0%	0%	0%	0%	0%	0%	0%
3.5%	58%	63%	18%	4%	1%	0%	0%	0%	0%	0%	0%	0%	0%	0%	0%	0%	0%	0%	0%
3.0%	68%	73%	23%	8%	2%	1%	0%	0%	0%	0%	0%	0%	0%	0%	0%	0%	0%	0%	0%
2.5%	77%	82%	40%	21%	4%	3%	1%	0%	0%	0%	0%	0%	0%	0%	0%	0%	0%	0%	0%
2.0%	81%	84%	55%	33%	11%	6%	4%	1%	0%	0%	0%	0%	0%	0%	0%	0%	0%	0%	0%
1.5%	89%	86%	69%	53%	24%	21%	12%	8%	4%	2%	1%	0%	0%	0%	0%	0%	0%	0%	0%
1.0%	92%	86%	91%	80%	57%	52%	34%	22%	8%	15%	6%	7%	6%	4%	0%	0%	0%	0%	0%
0.5%	100%	98%	98%	92%	92%	89%	80%	70%	55%	62%	34%	41%	24%	35%	62%	55%	46%	35%	28%
0.0%	0%	0%	0%	0%	0%	0%	0%	0%	0%	0%	0%	0%	0%	0%	0%	0%	0%	0%	0%

Supplementary File S3: Variation of Dataset 1. In the plots of A and B the SD on the y-axis is plotted against the AF (Reference value) on the x-axis (A: range 0-50%; B: range 0-10%) for each coverage (colours). Table C includes the actual values for the performance metric that was visualized in Figure 3A. In table D the interquartile ranges for each condition is indicated. In the tables, conditions with a false negative percentage greater than 75% were excluded. Note that a targeted AF of 0% and 100% corresponds to the wild-type and mutant respectively and is therefore not presented.

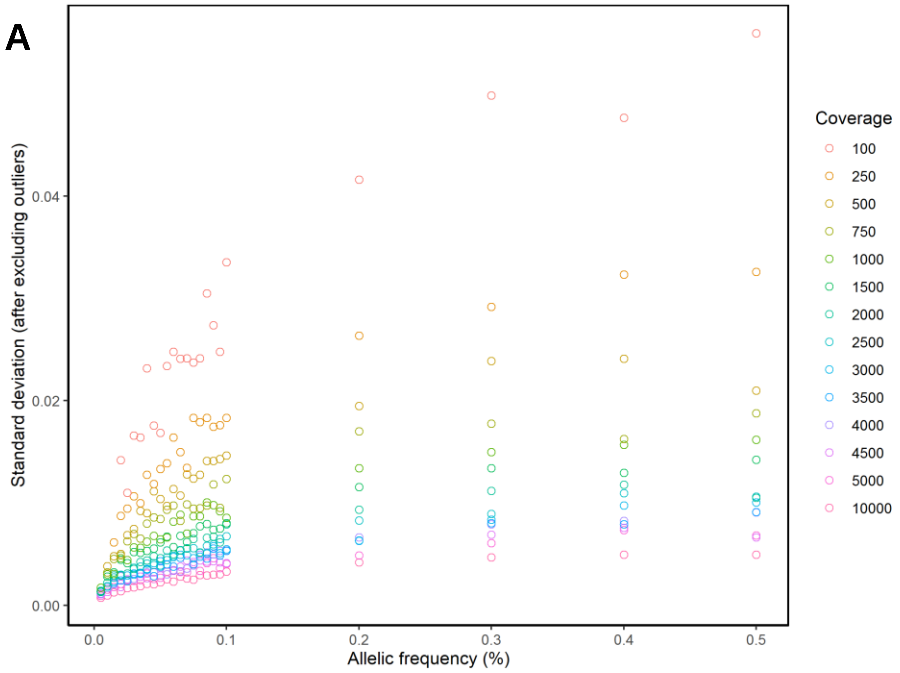

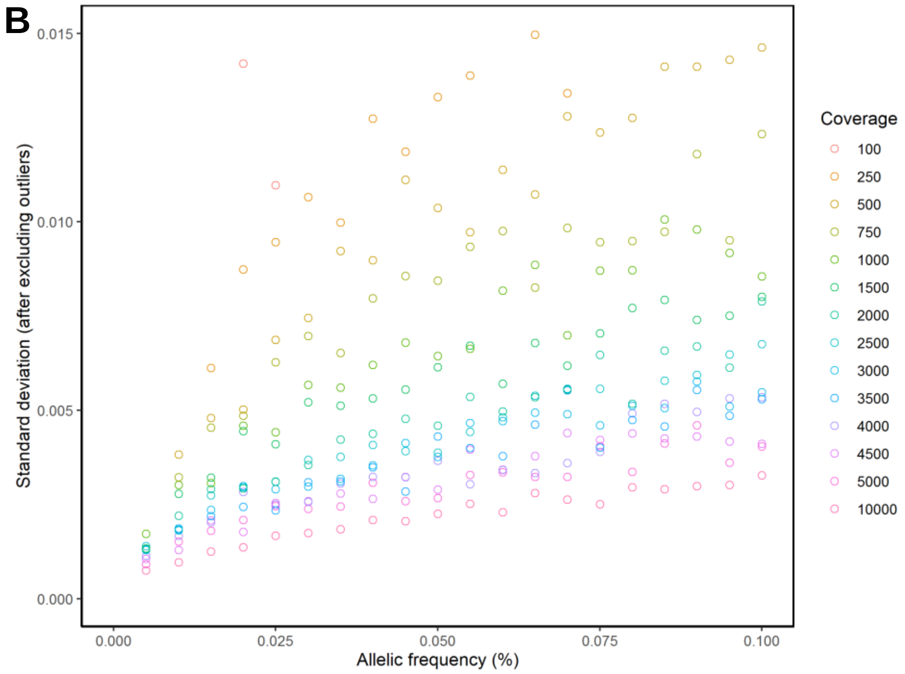

C

Coverage → AF ↓	100	250	500	750	1000	1500	2000	2500	3000	3500	4000	4500	5000	10000
**50.0%**	1.00	0.34	0.14	0.11	0.08	0.06	0.04	0.04	0.03	0.03	0.03	0.01	0.01	0.01
**40.0%**	1.00	0.46	0.26	0.12	0.11	0.07	0.06	0.05	0.04	0.03	0.03	0.03	0.02	0.01
**30.0%**	1.00	0.34	0.23	0.13	0.09	0.07	0.05	0.03	0.03	0.03	0.03	0.02	0.01	0.01
**20.0%**	1.00	0.40	0.22	0.17	0.10	0.08	0.05	0.04	0.02	0.02	0.03	0.03	0.01	0.01
**10.0%**	1.00	0.30	0.19	0.14	0.06	0.06	0.06	0.04	0.03	0.02	0.03	0.01	0.01	0.01
**9.5%**	1.00	0.50	0.33	0.15	0.14	0.09	0.06	0.07	0.04	0.04	0.05	0.03	0.02	0.01
**9.0%**	1.00	0.41	0.27	0.19	0.13	0.07	0.06	0.05	0.04	0.04	0.03	0.02	0.03	0.01
**8.5%**	1.00	0.36	0.21	0.10	0.11	0.07	0.05	0.04	0.03	0.02	0.03	0.02	0.02	0.01
**8.0%**	1.00	0.55	0.28	0.15	0.13	0.10	0.05	0.05	0.05	0.04	0.04	0.03	0.02	0.02
**7.5%**	1.00	0.60	0.27	0.16	0.13	0.09	0.07	0.06	0.04	0.03	0.03	0.03	0.03	0.01
**7.0%**	1.00	0.31	0.28	0.17	0.08	0.07	0.05	0.05	0.04	0.05	0.02	0.03	0.02	0.01
**6.5%**	1.00	0.39	0.20	0.12	0.14	0.08	0.05	0.05	0.04	0.04	0.02	0.02	0.02	0.01
**6.0%**	1.00	0.44	0.21	0.16	0.11	0.05	0.04	0.04	0.04	0.02	0.02	0.02	0.02	0.01
**5.5%**	1.00	0.35	0.17	0.16	0.08	0.08	0.05	0.04	0.04	0.03	0.02	0.03	0.02	0.01
**5.0%**	1.00	0.63	0.38	0.25	0.15	0.13	0.07	0.05	0.05	0.07	0.05	0.03	0.03	0.02
**4.5%**	1.00	0.46	0.40	0.24	0.15	0.10	0.07	0.05	0.06	0.03	0.03	0.03	0.02	0.01
**4.0%**	1.00	0.30	0.15	0.12	0.07	0.05	0.04	0.03	0.02	0.02	0.02	0.01	0.02	0.01
**3.5%**	1.00	0.37	0.32	0.16	0.12	0.10	0.07	0.05	0.04	0.04	0.03	0.03	0.02	0.01
**3.0%**	1.00	0.41	0.20	0.18	0.12	0.10	0.05	0.05	0.03	0.03	0.02	0.02	0.02	0.01
**2.5%**	1.00	0.74	0.39	0.33	0.16	0.14	0.08	0.08	0.07	0.05	0.05	0.05	0.05	0.02
**2.0%**	1.00	0.38	0.13	0.12	0.10	0.10	0.04	0.04	0.04	0.03	0.04	0.02	0.02	0.01
**1.5%**		1.00	0.61	0.55	0.25	0.28	0.23	0.20	0.15	0.13	0.11	0.12	0.09	0.04
**1.0%**			1.00	0.71	0.63	0.53	0.33	0.23	0.24	0.23	0.19	0.11	0.16	0.06
**0.5%**					1.00	0.59	0.59	0.65	0.56	0.57	0.44	0.38	0.28	0.19

D

Coverage → AF ↓	100	250	500	750	1000	1500	2000	2500	3000	3500	4000	4500	5000	10,000
**50.0%**	6.26%	4.10%	2.44%	2.06%	1.93%	1.79%	1.28%	1.39%	1.28%	0.97%	0.91%	0.88%	0.70%	0.62%
**40.0%**	5.86%	3.45%	2.37%	1.94%	1.98%	1.55%	1.33%	1.18%	1.17%	0.87%	1.00%	0.91%	0.79%	0.55%
**30.0%**	6.06%	3.40%	2.83%	2.02%	1.89%	1.36%	1.39%	1.21%	1.07%	0.97%	0.84%	0.88%	0.86%	0.55%
**20.0%**	4.59%	2.74%	2.26%	1.99%	1.47%	1.34%	1.05%	0.90%	0.89%	0.95%	0.73%	0.74%	0.67%	0.52%
**10.0%**	3.49%	2.47%	1.65%	1.33%	0.98%	0.85%	0.83%	0.86%	0.70%	0.59%	0.60%	0.50%	0.55%	0.36%
**9.5%**	3.03%	2.23%	1.81%	1.44%	1.00%	0.71%	0.80%	0.67%	0.74%	0.67%	0.56%	0.48%	0.42%	0.37%
**9.0%**	3.16%	2.11%	1.65%	1.27%	1.14%	0.91%	0.78%	0.73%	0.67%	0.62%	0.56%	0.55%	0.47%	0.34%
**8.5%**	3.50%	2.09%	1.63%	1.25%	0.90%	0.92%	0.69%	0.65%	0.61%	0.54%	0.62%	0.48%	0.45%	0.31%
**8.0%**	2.95%	1.81%	1.53%	1.24%	1.03%	0.87%	0.68%	0.56%	0.52%	0.47%	0.52%	0.54%	0.48%	0.32%
**7.5%**	3.12%	2.02%	1.55%	1.14%	0.96%	0.84%	0.74%	0.67%	0.60%	0.50%	0.59%	0.52%	0.51%	0.31%
**7.0%**	3.15%	2.26%	1.49%	1.16%	0.92%	0.80%	0.71%	0.53%	0.61%	0.62%	0.50%	0.48%	0.51%	0.29%
**6.5%**	2.38%	1.64%	1.42%	1.25%	0.90%	0.78%	0.59%	0.62%	0.53%	0.53%	0.41%	0.47%	0.40%	0.33%
**6.0%**	2.87%	1.87%	1.34%	1.17%	0.90%	0.73%	0.71%	0.55%	0.50%	0.51%	0.40%	0.44%	0.41%	0.26%
**5.5%**	2.47%	1.60%	1.06%	1.12%	0.84%	0.69%	0.59%	0.46%	0.48%	0.47%	0.48%	0.44%	0.39%	0.28%
**5.0%**	2.07%	1.55%	1.32%	0.97%	0.67%	0.70%	0.52%	0.55%	0.47%	0.51%	0.44%	0.39%	0.30%	0.27%
**4.5%**	2.41%	1.58%	1.15%	0.90%	0.87%	0.64%	0.58%	0.48%	0.46%	0.38%	0.37%	0.35%	0.30%	0.25%
**4.0%**	2.41%	1.42%	1.18%	0.92%	0.68%	0.58%	0.53%	0.47%	0.48%	0.42%	0.36%	0.38%	0.34%	0.25%
**3.5%**	1.81%	1.24%	1.04%	0.75%	0.73%	0.59%	0.55%	0.41%	0.38%	0.37%	0.40%	0.29%	0.32%	0.23%
**3.0%**	2.02%	1.28%	0.83%	0.86%	0.76%	0.67%	0.49%	0.42%	0.38%	0.38%	0.29%	0.30%	0.31%	0.20%
**2.5%**	2.08%	1.37%	0.78%	0.74%	0.59%	0.47%	0.37%	0.37%	0.29%	0.34%	0.33%	0.28%	0.30%	0.21%
**2.0%**	2.08%	1.13%	0.70%	0.56%	0.52%	0.41%	0.33%	0.37%	0.33%	0.29%	0.31%	0.27%	0.22%	0.20%
**1.5%**	1.65%	0.86%	0.57%	0.48%	0.37%	0.33%	0.36%	0.28%	0.28%	0.26%	0.25%	0.22%	0.22%	0.14%
**1.0%**	1.82%	0.92%	0.54%	0.39%	0.30%	0.25%	0.31%	0.22%	0.21%	0.22%	0.20%	0.17%	0.18%	0.11%
**0.5%**	1.16%	0.99%	0.60%	0.41%	0.33%	0.24%	0.17%	0.16%	0.14%	0.13%	0.14%	0.12%	0.10%	0.08%

Supplementary File S4: Variation of Dataset 2. In the plots of A and B the SD on the y-axis is plotted against the AF on the x-axis (A: range 0-50%; B: range 0-10%) for each coverage (colours). Table C includes the actual values for the performance metric that was visualized in Figure 3B. In table D the interquartile ranges for each condition is indicated, while excluding false negative results. In the tables, conditions with a false negative percentage greater than 75% were excluded. Note that a targeted AF of 0% and 100% corresponds to the wild-type and mutant respectively and is therefore not presented.

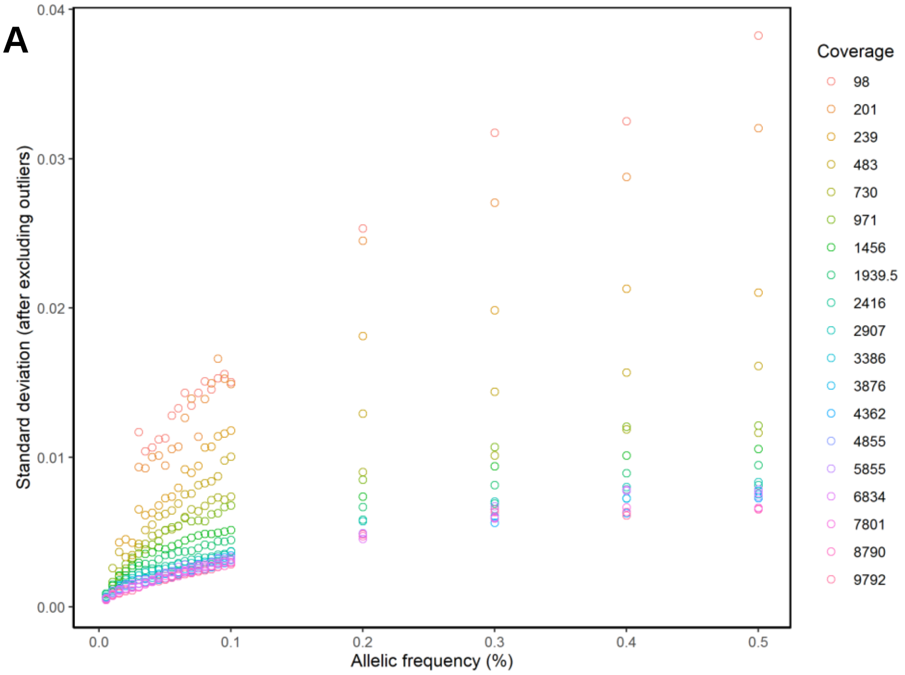

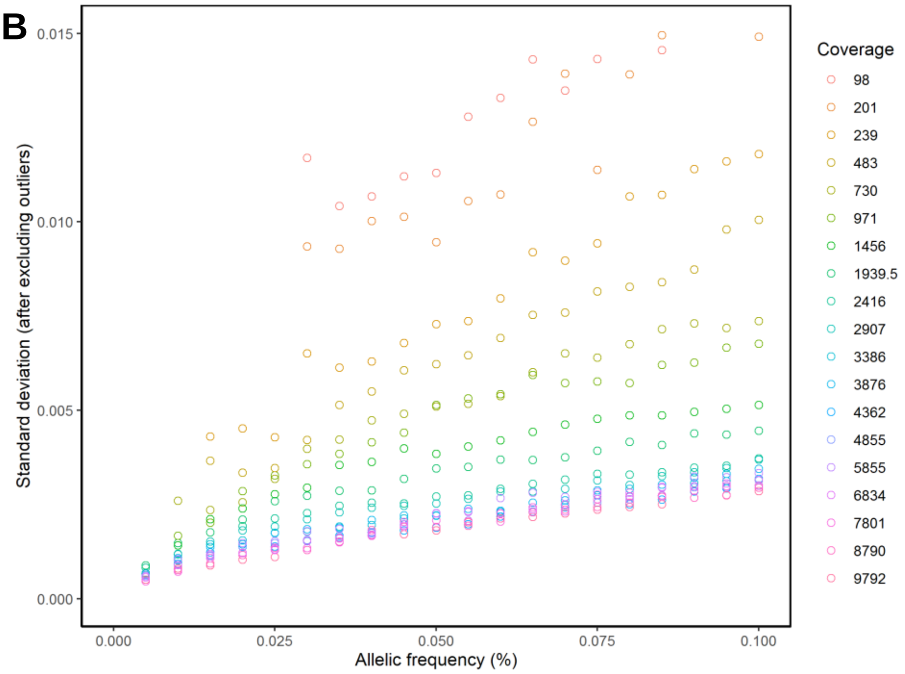

C

Coverage → AF ↓	97	201	237	482	728	969	1454	1937	2413	2904	3383	3872	4358	4851	5855	6834	7801	8790	9792
**50.0%**	1.00	0.70	0.30	0.18	0.09	0.10	0.08	0.06	0.05	0.05	0.04	0.04	0.04	0.04	0.04	0.04	0.03	0.03	0.03
**40.0%**	1.00	0.78	0.43	0.23	0.13	0.14	0.10	0.08	0.06	0.06	0.05	0.05	0.04	0.04	0.06	0.06	0.04	0.04	0.04
**30.0%**	1.00	0.73	0.39	0.21	0.10	0.11	0.09	0.07	0.05	0.05	0.04	0.04	0.03	0.03	0.05	0.04	0.03	0.04	0.04
**20.0%**	1.00	0.93	0.51	0.26	0.13	0.11	0.08	0.07	0.05	0.05	0.03	0.04	0.03	0.03	0.04	0.03	0.04	0.03	0.04
**10.0%**	1.00	0.98	0.62	0.45	0.24	0.20	0.12	0.09	0.06	0.06	0.04	0.05	0.04	0.04	0.05	0.04	0.04	0.04	0.04
**9.5%**	1.00	0.96	0.56	0.40	0.21	0.18	0.10	0.08	0.05	0.05	0.04	0.05	0.04	0.04	0.04	0.04	0.04	0.03	0.03
**9.0%**	0.85	1.00	0.47	0.28	0.19	0.14	0.09	0.07	0.04	0.04	0.03	0.04	0.03	0.03	0.04	0.03	0.03	0.03	0.03
**8.5%**	0.95	1.00	0.51	0.32	0.23	0.17	0.11	0.07	0.05	0.05	0.04	0.04	0.03	0.03	0.04	0.04	0.03	0.03	0.03
**8.0%**	1.00	0.85	0.50	0.30	0.20	0.14	0.10	0.08	0.05	0.04	0.03	0.04	0.03	0.03	0.03	0.03	0.03	0.03	0.03
**7.5%**	1.00	0.63	0.43	0.32	0.20	0.16	0.11	0.08	0.05	0.05	0.04	0.04	0.03	0.03	0.04	0.04	0.03	0.03	0.03
**7.0%**	0.94	1.00	0.41	0.30	0.22	0.17	0.11	0.07	0.05	0.04	0.03	0.04	0.03	0.03	0.04	0.03	0.03	0.03	0.03
**6.5%**	1.00	0.78	0.41	0.28	0.18	0.17	0.10	0.07	0.05	0.04	0.03	0.03	0.03	0.03	0.04	0.03	0.03	0.03	0.02
**6.0%**	1.00	0.65	0.36	0.27	0.16	0.17	0.10	0.08	0.05	0.05	0.03	0.03	0.03	0.03	0.04	0.03	0.02	0.03	0.03
**5.5%**	1.00	0.68	0.33	0.25	0.16	0.17	0.10	0.07	0.05	0.04	0.03	0.03	0.02	0.03	0.03	0.03	0.03	0.02	0.03
**5.0%**	1.00	0.70	0.42	0.30	0.20	0.21	0.12	0.09	0.06	0.05	0.04	0.04	0.03	0.03	0.04	0.04	0.03	0.03	0.03
**4.5%**	1.00	0.82	0.37	0.29	0.19	0.15	0.13	0.08	0.05	0.05	0.04	0.04	0.03	0.03	0.03	0.03	0.03	0.03	0.02
**4.0%**	1.00	0.88	0.35	0.27	0.20	0.15	0.12	0.07	0.06	0.05	0.03	0.04	0.03	0.03	0.03	0.03	0.02	0.03	0.02
**3.5%**	1.00	0.80	0.35	0.24	0.16	0.14	0.12	0.08	0.06	0.05	0.03	0.03	0.03	0.02	0.03	0.02	0.02	0.02	0.02
**3.0%**	1.00	0.64	0.31	0.13	0.12	0.09	0.06	0.05	0.04	0.03	0.02	0.02	0.02	0.02	0.02	0.02	0.01	0.01	0.01
**2.5%**			1.00	0.65	0.55	0.58	0.42	0.36	0.25	0.20	0.17	0.16	0.11	0.12	0.13	0.09	0.09	0.10	0.07
**2.0%**			1.00	0.55	0.32	0.40	0.28	0.22	0.18	0.16	0.12	0.10	0.09	0.09	0.11	0.07	0.07	0.07	0.05
**1.5%**			1.00	0.73	0.30	0.22	0.24	0.17	0.12	0.13	0.11	0.10	0.08	0.07	0.07	0.08	0.06	0.05	0.04
**1.0%**					1.00	0.41	0.30	0.32	0.21	0.21	0.15	0.17	0.13	0.16	0.16	0.12	0.08	0.09	0.10
**0.5%**								1.00	0.88	0.60	0.59	0.58	0.52	0.53	0.46	0.41	0.28	0.42	0.33

D

Coverage →<br>AF ↓	97	201	237	482	728	969	1454	1937	2413	2904	3383	3872	4358	4851	5855	6834	7801	8790	9792
**50.0%**	3.90%	4.30%	2.75%	1.94%	1.56%	1.53%	1.25%	1.07%	0.90%	0.89%	0.78%	0.84%	0.73%	0.77%	0.97%	0.86%	0.92%	0.96%	0.97%
**40.0%**	5.08%	5.67%	3.56%	2.27%	1.83%	1.78%	1.61%	1.16%	0.99%	0.99%	0.87%	0.89%	0.79%	0.82%	1.11%	1.33%	1.14%	0.93%	0.87%
**30.0%**	5.65%	6.02%	3.97%	2.35%	1.75%	1.82%	1.70%	1.36%	1.05%	1.04%	0.85%	0.91%	0.76%	0.82%	1.14%	0.95%	0.95%	1.18%	1.09%
**20.0%**	4.86%	6.93%	4.02%	2.73%	1.76%	1.52%	1.44%	1.30%	1.17%	1.08%	0.84%	0.78%	0.70%	0.72%	0.75%	0.70%	0.80%	0.77%	0.77%
**10.0%**	3.93%	5.28%	3.25%	2.44%	1.80%	1.65%	1.02%	0.83%	0.83%	0.81%	0.77%	0.77%	0.74%	0.65%	0.54%	0.49%	0.43%	0.42%	0.41%
**9.5%**	3.78%	5.68%	3.09%	2.44%	1.82%	1.67%	0.97%	0.79%	0.78%	0.78%	0.67%	0.74%	0.72%	0.66%	0.50%	0.50%	0.43%	0.40%	0.41%
**9.0%**	3.78%	5.65%	2.81%	2.18%	1.74%	1.63%	0.98%	0.77%	0.72%	0.82%	0.68%	0.68%	0.73%	0.66%	0.49%	0.47%	0.43%	0.42%	0.36%
**8.5%**	3.50%	5.39%	2.55%	2.41%	1.68%	1.64%	0.96%	0.78%	0.67%	0.78%	0.66%	0.65%	0.59%	0.64%	0.49%	0.48%	0.43%	0.44%	0.38%
**8.0%**	3.31%	5.34%	2.53%	2.23%	1.68%	1.67%	1.00%	0.80%	0.62%	0.68%	0.63%	0.61%	0.58%	0.62%	0.37%	0.44%	0.44%	0.40%	0.36%
**7.5%**	3.14%	4.05%	2.33%	1.98%	1.67%	1.57%	1.02%	0.80%	0.62%	0.71%	0.67%	0.62%	0.55%	0.54%	0.46%	0.43%	0.41%	0.41%	0.36%
**7.0%**	3.05%	4.32%	2.36%	2.44%	1.80%	1.41%	1.11%	0.75%	0.62%	0.61%	0.57%	0.55%	0.52%	0.56%	0.49%	0.36%	0.38%	0.38%	0.34%
**6.5%**	2.93%	4.17%	2.26%	2.73%	1.70%	1.42%	1.08%	0.75%	0.66%	0.57%	0.53%	0.58%	0.53%	0.50%	0.55%	0.36%	0.38%	0.38%	0.38%
**6.0%**	2.81%	4.30%	2.14%	2.50%	1.61%	1.32%	1.10%	0.77%	0.62%	0.53%	0.46%	0.51%	0.54%	0.49%	0.53%	0.42%	0.27%	0.35%	0.34%
**5.5%**	2.49%	2.94%	2.15%	2.24%	1.51%	1.62%	1.13%	0.81%	0.60%	0.57%	0.44%	0.51%	0.48%	0.44%	0.49%	0.46%	0.36%	0.32%	0.32%
**5.0%**	2.27%	1.95%	2.28%	2.02%	1.25%	1.44%	1.08%	0.89%	0.58%	0.54%	0.45%	0.43%	0.42%	0.45%	0.44%	0.46%	0.38%	0.34%	0.29%
**4.5%**	2.15%	1.70%	2.31%	1.75%	1.52%	1.22%	0.99%	0.87%	0.66%	0.52%	0.45%	0.40%	0.36%	0.43%	0.37%	0.40%	0.40%	0.34%	0.31%
**4.0%**	1.85%	1.55%	1.95%	1.51%	1.28%	1.25%	0.89%	0.88%	0.69%	0.53%	0.42%	0.43%	0.33%	0.35%	0.36%	0.33%	0.34%	0.36%	0.32%
**3.5%**	1.34%	1.68%	1.72%	1.33%	1.13%	1.33%	1.09%	0.75%	0.64%	0.62%	0.43%	0.39%	0.36%	0.34%	0.38%	0.31%	0.29%	0.33%	0.34%
**3.0%**	1.48%	1.18%	1.51%	1.15%	0.99%	1.26%	0.80%	0.67%	0.61%	0.58%	0.47%	0.41%	0.34%	0.34%	0.32%	0.32%	0.27%	0.27%	0.29%
**2.5%**			0.87%	1.09%	0.82%	1.04%	0.75%	0.72%	0.55%	0.54%	0.45%	0.44%	0.37%	0.31%	0.28%	0.24%	0.26%	0.28%	0.23%
**2.0%**			0.96%	0.99%	0.71%	0.74%	0.81%	0.65%	0.54%	0.45%	0.43%	0.44%	0.35%	0.36%	0.28%	0.22%	0.24%	0.23%	0.22%
**1.5%**			0.50%	0.94%	0.71%	0.57%	0.59%	0.56%	0.38%	0.39%	0.40%	0.35%	0.32%	0.33%	0.29%	0.30%	0.23%	0.18%	0.19%
**1.0%**					0.55%	0.48%	0.37%	0.36%	0.34%	0.37%	0.31%	0.30%	0.25%	0.28%	0.18%	0.21%	0.24%	0.19%	0.22%
**0.5%**								0.20%	0.18%	0.22%	0.16%	0.18%	0.16%	0.18%	0.23%	0.23%	0.20%	0.10%	0.14%
